# Effect of aerobic training on hot flushes and quality of life—a randomized controlled trial

**DOI:** 10.3109/07853890.2011.583674

**Published:** 2011-06-03

**Authors:** Riitta Luoto, Jaana Moilanen, Reetta Heinonen, Tomi Mikkola, Jani Raitanen, Eija Tomas, Katriina Ojala, Kirsi Mansikkamäai, Clas-Håkan Nygård

**Affiliations:** 1 UKK Institute for Health Promotion Research, Tampere, Finland; 2 National Institute for Health and Welfare, Helsinki, Finland; 3 School of Health Sciences, University of Tampere, Finland; 4 Helsinki University Central Hospital, Helsinki, Finland, and; 5 Tampere University Central Hospital, Tampere, Finland

**Keywords:** Aerobic training, exercise, hot flushes, quality of life, randomized controlled trial

## Abstract

*Background and objective*. To estimate whether aerobic training has an effect on frequency of hot flushes or quality of life.

*Design*. A randomized controlled trial.

*Participants and setting*. Symptomatic, sedentary women (*n* = 176), 43–63 years, no current use of hormone therapy.

*Intervention*. Unsupervised aerobic training for 50 minutes four times per week during 6 months.

*Outcomes*. Hot flushes as measured with Women's Health Questionnaire (WHQ) and Health-Related Quality of Life (HRQoL, SF-36), daily reported hot flushes on phone-based diary, cardiorespiratory fitness (CRF), and body composition.

*Results*. Intervention group had larger decrease in the frequency of night-time hot flushes based on phone diary (*P* for month X group = 0.012), but not on WHQ scale. Intervention group had less depressed mood (*P*= 0.01) than control women according to change in WHQ score. Changes in WHQ score in depressed mood (*P* = 0.03) and menstrual symptoms (*P*=0.01) in the intervention group were significantly dependent on frequency of training sessions. HRQoL was improved among the intervention group women in physical functioning (*P*= 0.049) and physical role limitation (*P*= 0.017). CRF improved (*P*= 0.008), and lean muscle mass increased (*P*= 0.046) significantly in the intervention group as compared to controls.

*Conclusions*. Aerobic training may decrease the frequency of hot flushes and improve quality of life among slightly overweight women.

## Introduction

Hot flushes (i.e. vasomotor symptoms) are a common complaint in many women and may occur for years beyond menopausal transition (1–3). Hormonal and thermoregulatory involvement in the occurrence of hot flushes is likely, and many forms of pharmacological as well as non-pharmacological alleviation have been studied (4). Although traditional post-menopausal hormone therapy (HT) is the most effective treatment to alleviate menopausal hot flushes, it has been subject to major criticism due to the results of large clinical trials in which the risks of HT have outweighed the benefits (5). Women with higher adiposity report more severe hot flushes (6), and successful weight loss has been associated with hot flush decrease in a randomized trial (7). Because increased physical activity has beneficial effects on weight management, it could be one alternative treatment to HT in alleviating menopausal symptoms. Furthermore, physical exercise is also known to increase hypothalamic β -endorphin production, which may stabilize thermoregulation known to be disturbed during menopausal hot flushes (8).

**Key messages**

- Aerobic training may decrease the frequency of hot flushes.
- Aerobic training four times per week improved quality of life among symptomatic women.
- Quality of life improvement was dependent on frequency of training sessions.

Evidence concerning whether or not physical training reduces hot flushes is still inconclusive. Some studies have suggested that increasing physical activity may possibly decrease the number of hot flushes (6), but studies showing no association (9) or an inverse association (10,11) have been published as well. Previous randomized clinical trials on exercise and hot flush reduction have either had small or selected populations, symptom reduction as a non-primary outcome, or other limitations such as non-symptomatic women or lack of a control group (12–16). Our objective was to estimate whether aerobic training decreases hot flushes or increases health-related or menopause-specific quality of life among symptomatic menopausal women.

## Material and methods

### Study design and participants

With the approval of Pirkanmaa Hospital District Ethics Committee, we recruited healthy Caucasian women via newspaper advertisements in Pirkanmaa area between January and March 2009. Women who were willing to participate to the trial after reading the newspaper advertisement were screened for inclusion criteria through a telephone interview. If they were eligible and willing to participate, they received information concerning the trial by mail. Participants were not paid for being included, neither in intervention nor control group, since this is not allowed in medical studies performed in Finland. All participants gave written informed consent.

**Figure 1:**
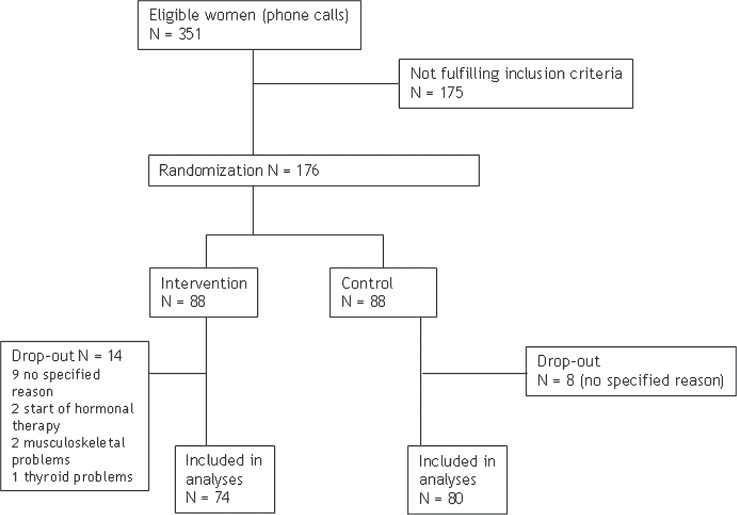
Participant flow in a randomized aerobic training trial.

Design was randomized controlled study with allocation ratio of one. Women were assigned to intervention and control groups by computer randomization. Envelopes including information on assigned group were delivered by research nurses. Inclusion criteria for the study were: symptomatic (daily hot flushes), age 40–63 years, no current use or use in the past 3 months of HT, sedentary (aerobic training less than twice weekly), and 6–36 months since last menstruation (Figure 1). Women who were physically active (exercising ≥2 times/week, for at least 30 minutes each time), or whose body mass index (BMI) was over 35 kg/m^2^, or who had coronary heart disease, or orthopaedic or other diseases preventing them from exercising, were excluded from the study. In addition, women who were using medication influencing heart rate (β-blockers, sympathomimetics) were excluded, since they would have biased the results concerning heart rate. Menopausal status was verified by assay of plasma follicle-stimulating hormone (FSH), which had to exceed 30 IU/L.

Before initiating the trial, recruited women kept a 2-week diary (pencil and paper) concerning day-time and night-time hot flushes. The women were instructed to record as a hot flush a sensation of warmth, with or without concomitant sweating. To obtain an estimate of the overall impact of hot flushes we calculated weekly and daily frequency of all hot flushes before the intervention, and daily frequency of hot flushes both day-time and night-time during the intervention. From a total of 351 women, 176 women participated in the study; 175 women were excluded since they did not meet the inclusion criteria. The duration of intervention was 6 months.

### Outcomes

The primary outcome measure of the study was hot flushes as measured with Women's Health Questionnaire (WHQ). Secondary outcomes to be reported in this paper were hot flushes reported by phone-based diary, health-related quality of life as measured with SF-36 scale, cardiorespiratory fitness (as estimated by the Urho Kekkosen Kuntoinstituuttisäätiö (UKK) 2 km Walk Test (17)), and body composition (weight, fat and lean mass; kg). Specific questions related to sleep will be reported in a separate article.

The WHQ was used as an instrument to assess menopause-specific quality of life (18). The questionnaire has been validated in the Finnish language, and test-retest reliability has been evaluated with a 2-week interval (19). It is composed of 36 items covering mood states, physical sensations and experience, sexual behaviour, and hot flushes, with a total of nine sub-scales (depressed mood, somatic symptoms, hot flushes, anxiety/fears, sexual behaviour, sleep problems, menstrual symptoms, memory/concentration, attractiveness). Self-reported symptoms are scored on a five-point scale (values from 0 to 4).

Health-related quality of life was measured by using the SF-36 Health Survey questionnaire, which has been validated in Finland (20) and is known to be one of the most reliable and widely used quality of life questionnaires. It is composed of 36 items assessing eight dimensions of quality of life: physical functioning, physical role limitation, bodily pain, general health, vitality, social functioning, emotional role limitation, and mental health. For each of the eight domains scores were transformed linearly to a scale ranging from 0 (maximal impairment) to 100 (no impairment, best quality of life).

### Measurements

Information on day-time and night-time hot flushes and aerobic training was collected by using mobile phone questionnaires twice a day. The mobile phone questionnaire included pre-specified questions on hot flushes, sleep, and other symptoms. During the morning, the structured-item questionnaire included five specific questions on hot flushes (yes/no), question on night sweating (yes/no), and two questions on sleep quality (yes/no). During the evening, the structured-item questionnaire included the same questions as in the morning questionnaire, with added (yes/no) questions regarding headache, mood swings, dysphoria, depression, dryness of the vaginal mucosa, and urinary and other symptoms. If the woman did not want to use her own mobile phone, or the phone did not have a 3G technology option, the phones were arranged by the study team. Responses were automatically transferred via 3G technology into digital format. Since the symptom diary method had not been used earlier, it was evaluated by way of a separate usability questionnaire (SUS; system usability scale (21)) immediately after 6 months of intervention. Most of the women participating in the intervention also responded to the usability questionnaire (response rate 72.7%). The score in the usability questionnaire was 75.4 (range 0 – 100). The women reported the proportions of both day-time and night-time hot flushes, which were compulsory information programmed to the phone. Reporting on severity of hot flushes during the trial was an option, not compulsory, and thus lacking in half the responses, although at base-line hot flush status was collected from all women using conventional methods (paper).

Base-line questionnaires (on paper) included information on employment, life-style, dietary habits, and other health-related issues. The women also received a questionnaire on life-style changes at the 12th week and at the end of the trial (24 weeks). Although the phone-based questionnaire was not validated, the questions used were simple (yes/no) and expressed the existence of symptoms without grading them.

Fat and lean mass and body mass index were measured by dual X-ray absorptiometry (DXA). Body mass index was calculated as the ratio of weight (kg) to height squared (m^2^). Cardiorespiratory fitness was estimated by using the UKK Walk Test, which is a validated method for measuring aerobic fitness of 20–65-year-old adults who have no illnesses or disabilities that limit brisk walking and who are not on medication that affects heart rate (17). Heart rate was monitored during the walk and registered immediately at the end (Polar Electro, M61, Finland). Measurements of cardiorespiratory fitness (CRF) were performed twice at base-line due to the effect of learning.

### Aerobic training

Women in the intervention group kept physical activity diaries once a week during the first month; thereafter once a month. Women in the control group were asked (via questionnaire on paper) once in the middle of the trial (11–12 weeks from base-line) whether they had changed any of their physical activities or dietary habits.

The unsupervised aerobic training programme included aerobic training four times per week, with 50 minutes of exercise each time. Ratings of perceived exertion (RPE) were used to check the intensity of aerobic training (22). Participants were instructed to exercise at a level corresponding to 13–16 on the scale from 6 to 20. This corresponds to about 64%–80% of maximal heart rate (22). At least two sessions had to include walking or Nordic walking, and the other two sessions could include walking, Nordic walking, jogging, cycling, swimming, skiing, aerobics, or other gymnastic exercise. The intervention mainly involved walking, because earlier UKK trials have shown favourable results on health among menopausal and post-menopausal women (23). Adherence to the trial was supported by an option to participate in instructed aerobics or step aerobics sessions at the UKK Institute twice a week.

The intervention group wore heart rate monitor belts (Suunto®; Memory Belt, Suunto, Vantaa, Finland) in training sessions. Every second week an aerobic training instructor gave feedback to the participants concerning the training sessions. Data collected from the heart rate belts were transferred to a computer and analysed with a software program, Firstbeat technologies HEALTH (Firstbeat Health®, Finland). Participants also reported their exercise by mobile phone and received weekly feedback by program message and an additional program message in case responses were not received during 4 days. Frequency of realized aerobic training sessions was based on both heart rate monitor belt information (sessions lasting at least 15 minutes and at most 4 hours, with at most 20% error) and phone-based aerobic training diary.

Both the intervention and control groups attended lectures once or twice per month given by the principal investigator of the study (R.L.). The lectures took 60–75 minutes and mostly covered topics of physical activity and general health. The women were not compensated financially for participation in the trial, but expenses arising from the use of mobile phones were covered. Aerobic training-based injuries were monitored by means of questionnaires, and no important harms or unintended effects occurred.

### Statistical analysis

Base-line characteristics of the groups were tabulated (means and standard deviations, or percentages; Table I). Differences at base-line between the randomized groups were evaluated by using Student's *t* test for continuous variables when they were normally distributed, and the Mann–Whitney *U* test when non-normally distributed. Normality was evaluated with the Kolmogorov–Smirnov test. Categorical variables were tested using the chi-square test. All analyses were performed in originally assigned groups. Missing values concerning the primary outcome (WHQ) were imputed by using base-line value in case a 6-month value was missing; 22 persons had imputed values in 1–9 items of WHQ. Multivariate modelling was not used since the study was a randomized trial and there were no differences in base-line confounding factors between the groups.

**Table I:** Base-line characteristics and quality of life in the intervention and control groups. QoL was estimated by means of the Short Form-36 quality of life (SF-36) score and the menopause-specific quality of life score (Women's Health Questionnaire (WHQ)).

	Intervention *n* = 74	Control *n* = 77	*P* for difference^a^	Missing (intervention, control)
Age, y	54.5±3.8	54.2±3.7	0.73	−
University degree, %	24.3	26.0	0.82	−
Employed, %	83.6	77.6	0.36	1, 1
Smoker or occasional smoker, %	18.1	11.7	0.27	2, 0
Weight, kg	70.5 ±11.4	71.7±12.5	0.56	−
Body mass index, kg/m^2^	26.3±4.0	26.9±4.3	0.42	−
Follicle-stimulating hormone (FSH), mmol/L	65.4±21.2	60.2±24.1	0.16	−
Thyroid-stimulating hormone (TSH), mmol/L	2.40±1.96	2.72±1.82	0.28	−
Weekly frequency of hot flushes, %				
<29.5	31.9	37.8		
30–49.5	31.9	23.0		
>50	36.2	39.2	0.48	5, 3
Health-related quality of life				
SF-36 physical component score				
Physical functioning	90 (20–100)	90 (40–100)	0.39	−
Role–physical	100 (0–100)	100 (0–100)	0.44	1, 1
Bodily pain	67.5 (0–100)	67.5 (22.5–100)	0.30	1, 0
General health	65 (10–95)	65 (25–100)	0.72	1, 0
SF-36 mental component score				
Vitality	65 (5–95)	60 (0–90)	0.28	−
Social functioning	87.5 (25–100)	87.5 (12.5–100)	0.33	1, 0
Role–emotional	100 (0–100)	100 (0–100)	0.60	1, 1
Mental health	76 (4–100)	76 (24–100)	0.46	−
Menopause-specific quality of life				
Depressed mood	0.14 (0–0.86)	0.14 (0–0.86)	0.18	−
Somatic symptoms	0.43 (0–1)	0.43 (0–0.86)	0.84	2, 1
Memory/concentration	0.67 (0–1)	0.67 (0–1)	0.44	1, 0
Vasomotor symptoms	1.00 (0–1)	1.00 (0–1)	0.54	2, 1
Anxiety/fears	0.13 (0–1)	0.25 (0–0.75)	0.59	−
Sexual behaviour	0.33 (0–1)	0.33 (0–1)	0.63	15, 26
Sleep problems	0.33 (0–1)	0.67 (0–1)	0.30	0, 0
Menstrual symptoms	0.33 (0–1)	0.33 (0–1)	0.73	2, 1
Attractiveness	0.00 (0–1)	0.00 (0–1)	0.53	3, 1

a Differences between groups were tested by Student's *t* test (continuous variables, normal distributions), the Mann–Whitney *U* test (continuous variables, non-normal distributions), or the chi-square test (categorical or dichotomized variables). Values are shown as means ± SD, medians and ranges, or percentages.

As an evaluation of dose-response effect, realized aerobic training sessions with sufficient intensity and duration were divided to three equal-size groups (low, medium, high) to enable analysis of change in WHQ and SF-36 scores from base-line to 6 months. The effect of realized aerobic training on between-group differences in WHQ and SF-36 was tested by use of non-parametric methods (Kruskal–Wallis test), because the distributions were not normal.

The groups had different proportions of women responding to the question concerning hot flushes yes or no—the proportions developed differently by the group during intervention time. Regression slopes were statistically compared by using mixed models for binary outcome (yes/no hot flushes).

Because the responses for the same person are not conditionally independent, a multilevel mixed-effect logistic regression model was fitted. This model allows for a difference between groups at base-line and linear changes in the log odds of hot flushes over time. To estimate the intervention effect, the interaction term (month X group) was included in the model.

The proportion of women with hot flushes was expected to be at least 68% (proportion among controls, P_c_). The expected difference between intervention and control group in proportion of women with hot flushes was at least 30%. Based on these assumptions, the proportion of women with hot flushes would be 47.3% among women in the intervention group. If alpha is 0.05 and beta 80%, the number needed in the sample is calculated with the following formula: *n* = (alpha + beta)^2^ X {[P_c_ X (1–P_c_)+ P_i_ X (1–P_i_)]/(P_c_–P_i_)^2^} = 89.7. Thus, 90 women were needed in each group to achieve 80% power.

## Results

### Base-line

Of the 176 women who started, 159 continued until the end of the study (90% compliance rate). Finally, 154 women (74 in the intervention group, 80 in the control group) completed the UKK Walk Test; 4 (3 in the intervention group, 1 in the control group) were not willing to complete this test. Dropouts' (*n* = 22; 14 in the intervention group, 8 in the control group) main reasons to discontinue the trial were personal reasons (*n* =17) and chronic illness (*n*= 3) or initiation of hormone replacement therapy (*n*=2).

Drop-out women whose base-line characteristics were available (*n*= 17, missing 5) were compared with responders (*n*= 159) in regard to age, education, employment status, smoking, weight, BMI, and FSH. Significant differences were found in age and weight—mean age of the respondents was 54.2 years (SD 3.7) and among drop-outs 51.1 years (SD 3.4) (*P*=0.001 in *t* test). Weight among drop-outs was on average 59.3 kg (SD 6.0) and among responders 71.9 kg (SD 12.5) (*P*= 0.045 in *t* test).

The participating women were on average 54 years of age, with a body mass index of 26–27 kg/m^2^ (Table I). There were no significant differences by group in any base-line values (Table I). At base-line, the mean number of all hot flushes (HF) per day independent of severity (mild, medium, or severe HF) among intervention women was 6.3 and among control women 5.9. Mean number of daily mild HF was 1.7 among the intervention women, 1.9 among controls; mean number of medium daily HF 2.9 and 2.8; and mean number of severe daily HF 1.6 and 1.0, respectively. The lowest number of HF a subject had at base-line was 0.11 HF/day. Average time for menopause was 4.5 years among intervention group women and 3.4 years among control women (SD 4.4 and 4.1, respectively) (*P* = 0.097).

Response rates did not differ between the exercise group and the control group: morning questionnaire, intervention group 74.4%, control group 69.4%; evening questionnaire, 69.8% and 70%, respectively.

### Hot flushes

There were no significant between-group differences in hot flushes according to WHQ (Table II).

**Table II:** Women's Health Questionnaire and SF-36 health-related quality-of-life scores among the intervention and control groups—6 month intervention scores and change from base-line to end.

	End intervention	Control	*P*^a^	Change intervention	Control	*P*^a^
Health-related quality of life						
SF-36 physical component score						
Physical functioning	89.0 (13.5)	87.1 (14.8)	0.33	2.67 (10.8)	–0.64 (9.38)	0.049
Role–physical	81.4 (29.3)	77.3 (33.5)	0.48	5.14 (27.9)	–3.95 (30.0)	0.017
Bodily pain	69.4 (21.1)	70.2 (24.9)	0.65	0.72 (19.0)	–3.08 (21.4)	0.32
General health	66.9 (21.1)	65.1 (20.0)	0.55	3.01 (12.3)	–0.45 (12.1)	0.065
SF-36 mental component score						
Vitality	66.9 (18.9)	58.3 (23.8)	0.023	5.72 (14.2)	–0.13 (16.3)	0.062
Social functioning	86.0 (19.6)	79.9 (25.1)	0.17	3.25 (19.1)	1.62 (20.9)	0.82
Role–emotional	85.6 (27.1)	78.4 (31.9)	0.12	6.85 (25.4)	3.07 (34.9)	0.46
Mental health	78.1 (15.9)	72.7 (17.6)	0.021	3.51 (11.9)	0.25 (13.2)	0.097
Menopause-specific quality of life score (WHQ)						
Depressed mood	0.12 (0.20)	0.22 (0.21)	<0.001	–0.07 (0.16)	–0.02 (0.15)	0.023
Somatic symptoms	0.42 (0.30)	0.41 (0.26)	0.96	–0.04 (0.23)	–0.02 (0.17)	0.64
Memory/concentration	0.49 (0.35)	0.50 (0.38)	0.84	–0.03 (0.30)	–0.07 (0.34)	0.53
Vasomotor symptoms	0.83 (0.32)	0.85 (0.28)	0.92	–0.10 (0.37)	–0.05 (0.25)	0.30
Anxiety/fears	0.17 (0.26)	0.19 (0.22)	0.25	–0.04 (0.16)	–0.02 (0.17)	0.82
Sexual behaviour	0.38 (0.35)	0.43 (0.34)	0.39	–0.02 (0.27)	–0.02 (0.24)	0.94
Sleep problems	0.37 (0.33)	0.45 (0.30)	0.072	–0.08 (0.28)	–0.05 (0.26)	0.28
Menstrual symptoms	0.26 (0.29)	0.31 (0.32)	0.32	–0.08 (0.25)	–0.05 (0.29)	0.47
Attractiveness	0.17 (0.30)	0.30 (0.39)	0.041	–0.10 (0.31)	–0.01 (0.28)	0.062

a *P* for difference between the groups; Mann—Whitney test.

**Figure 2:**
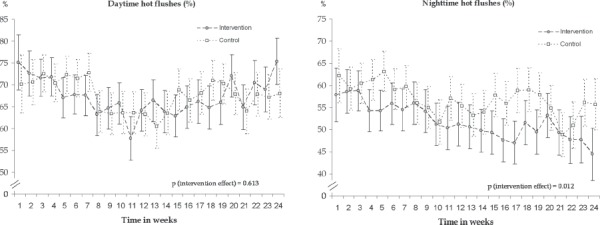
Observed weekly frequencies (%) and 95% confidence intervals of day-time (left) and night-time (right) hot flushes based on mobile phone questionnaire during 24 weeks of intervention.

At base-line phone-based diary, 57% of the women in the intervention and 61% of the women in the control group reported night-time hot flushes; proportions for day-time hot flushes were 72% and 71% (Table III). Decrease in day-time hot flushes was 2.9% in the intervention group (from 72.4% to 69.5%) and 4.5% (from 71.1% to 66.6%) in the control group. According to multilevel mixed models, the decrease by month in the number of nighttime (*P* for interaction group X month = 0.012) but not day-time hot flushes (*P* for interaction group X month = 0.61) was significantly larger in the intervention group than in the control group (Table III; Figure 2).

**Table III:** Number of responses to phone-based diary and observed frequency (%) of hot flushes by intervention month and group. Estimated proportion of hot flushes from multilevel mixed logistic regression.

	Intervention group				Control group			
		
Month	Responses	Number of responses with hot flushes	Observed proportion of responses with hot flushes (%)	Estimated proportion of hot flushes from mixed model	Responses	Number of responses with hot flushes	Observed proportion of responses with hot flushes (%)	Estimated proportion of hot flushes from mixed model
Night-time hot flushes:								
1 (base-line)	1513	867	57.3	56.1	1518	920	60.6	59.7
2	1719	948	55.1	54.3	1712	1018	59.5	58.6
3	1629	843	51.7	53.2	1576	863	54.8	58.1
4	1611	795	49.3	52.6	1581	872	55.2	55.6
5	1552	780	50.3	48.5	1503	866	57.6	54.3
6 (end)	1314	624	47.5	46.0	1361	716	52.6	54.0
Mixed model for day-time hot flushes:								
*P* (group at base-line) = 0.88.								
*P* (month) = <0.001.								
*P* (group X month) = 0.012.								
Day-time hot flushes:								
1 (base-line)	1314	951	72.4	68.2	1339	952	71.1	68.5
2	1626	1080	66.4	67.1	1640	1151	70.2	69.1
3	1537	970	63.1	66.5	1542	978	63.4	67.8
4	1495	964	64.5	67.2	1446	936	64.7	67.3
5	1386	931	67.2	66.2	1463	1014	69.3	66.6
6 (end)	1208	840	69.5	66.6	1292	861	66.6	65.7

Mixed model for day-time hot flushes:

*P* (group at base-line) = 0.18.

*P* (month) = 0.005.

*P* (group X month) = 0.61.

However, in day-time hot flushes there was a decrease until the 3-month point (Figure 2).

### Changes in WHQ and SF-36

Between-group differences in WHQ were significant in depressed mood (*P*< 0.001) and attractiveness (*P*= 0.04) and close to significant in sleep problems (*P*= 0.07) at the end of the trial. Between-group difference in change from base-line to 6 months was significant in depressed mood (*P*=0.02) and close to significant in attractiveness (*P*=0.06) (Table II). WHQ scores were significantly correlated with frequency of realized aerobic training sessions in depressed mood (*P*= 0.03) and menstrual symptoms (*P*=0.01) and close to significant in anxiety (*P*= 0.06) (Table IV). No differences were found in any of the WHQ items by base-line hot flush score (not shown in the table).

**Table IV:** Changes in quality of life (WHQ and SF-36 scores) by frequency of aerobic training sessions (from phone-based diary and heart rate belt information). Intervention group women (*n* = 78). Significance between the frequency groups tested with non-parametric Kruskal–Wallis test.

Change (base-line to 6 month) in:	Low (1–49 sessions) *n* = 24	Medium (50–72 sessions) *n* = 24	High (> 72 sessions) *n* = 26	*P*
SF-36	Mean (SD)	Mean (SD)	Mean (SD)	
Physical functioning	2.34 (11.3)	2.13 (13.2)	3.46 (8.0)	0.80
Role–physical	0.00 (22.1)	6.25 (28.8)	9.00 (32.2)	0.58
Bodily pain	3.04 (12.4)	–4.58 (19.1)	3.56 (22.9)	0.21
General health	0.83 (11.6)	0.63 (13.1)	7.40 (11.4)	0.096
Vitality	9.44 (13.0)	5.35 (15.9)	2.63 (13.4)	0.27
Social functioning	1.56 (23.4)	0.00 (16.5)	8.00 (16.5)	0.22
Role–emotional	6.94 (31.1)	5.56 (16.1)	8.00 (27.7)	0.99
Mental health	2.71 (12.6)	3.50 (11.9)	4.27 (11.7)	0.95
WHQ				
Depressed mood	0.01 (0.17)	–0.12 (0.16)	–0.09 (0.14)	0.03
Somatic symptoms	–0.04 (0.23)	0.04 (0.23)	–0.11 (0.20)	0.12
Memory/concentration	–0.03 (0.34)	–0.06 (0.25)	–0.02 (0.31)	0.85
Vasomotor symptoms	–0.04 (0.36)	–0.15 (0.35)	–0.13 (0.40)	0.52
Anxiety/fears	0.02 (0.07)	–0.05 (0.16)	–0.08 (0.20)	0.056
Sexual behaviour	–0.03 (0.22)	–0.03 (0.28)	–0.01 (0.32)	0.98
Sleep problems	–0.03 (0.17)	–0.11 (0.21)	–0.10 (0.41)	0.46
Menstrual symptoms	0.05 (0.23)	–0.16 (0.26)	–0.12 (0.20)	0.01
Attractiveness	–0.13 (0.22)	–0.04 (0.44)	–0.13 (0.22)	0.38

Missing number of responses: low: SF-36 1, WHQ 5; medium: SF-36 0, WHQ 5; high: SF-36 4, WHQ 16.

After 6 months' training, intervention group women had significantly higher SF-36 scores in vitality (*P*= 0.02) and mental health (*P*= 0.02) than did control women (Table II). Between-group differences in changes of SF-36 scores were significant in the areas of physical functioning (*P*= 0.049) and physical role limitation (*P*= 0.017) and close to significant in general health (*P*=0.07) and vitality (*P*=0.06) (Table II). Women with hot flush score higher than or equal to 78.5 (mean hot flush score at base-line) had significantly larger improvement in SF-36 vitality (*P*= 0.042) and mental health (*P*= 0.017) than did women with lower hot flush score (<78.5) at base-line. Changes in SF-36 score were not significantly related to frequency of aerobic training sessions (Table IV).

### Fitness and body composition

The groups did not differ as regards cardiorespiratory fitness or body composition at base-line (Table V). The women in the intervention group increased their estimated maximal oxygen consumption (VO_2_ max) statistically significantly in comparison with the control group (*P*= 0.008). Increase in lean mass was significantly higher among intervention group (0.57 kg versus 0.15 kg; *P*= 0.046) than among control group (Table V).

**Table V:** Aerobic training-related variables (fitness and body composition) at base-line, after 6 months intervention, and change between base-line and 6 months. Differences between groups were tested by *t* test.

	Base-line intervention	Control	*P*	End intervention	Control	*P*	Change intervention	Control	*P*
VO_2_ (mL/kg/min)	31.7 (5.4)	31.5 (4.8)	0.845	32.6 (5.2)	31.4 (5.2)	0.167	0.77 (2.22)	–0.16 (1.83)	0.008
DXA									
fat mass (kg)	27.2 (8.8)	28.3 (8.9)	0.413	26.1 (8.6)	27.8 (8.8)	0.262	–0.90 (1.99)	–0.48 (2.10)	0.219
lean (muscle mass, kg)	40.1 (4.3)	40.2 (4.9)	0.917	40.6 (4.1)	40.3 (4.6)	0.705	0.57 (1.16)	0.15 (1.34)	0.046

Missing values: Intervention: base-line 2, 0, and 0; end: 10, 5, and 5; change: 11, 5, and 5; Control: base-line–; end: 4, 3, and 3; change: 4, 3, and 3. DXA = dual X-ray absorptiometry; VO_2_ = volume of oxygen consumption.

### Other results

One woman in the intervention group and three women in the control group used SSRI/SNRI medication during the trial (difference not significant). Seven women in the intervention group and four women in the control group used natural remedies. When users of SSRI/SNRI and/or natural remedies were excluded from the analyses, change in SF-36 physical functioning was no more significant (*P*= 0.085) (Table II), and significance of change in lean muscle mass decreased as well (*P*= 0.066) (Table V). There were no users of excitatory drugs among either group, and the use of liquids including caffeine did not differ between the groups (mean number of cups of coffee 3.93, SD 1.88 intervention; 3.95, SD 1.94 control)

## Discussion

In our trial the intervention group had significantly larger decrease in the frequency of night-time hot flushes based on mobile phone diary, although in the WHQ scale between-group differences in hot flushes were not found. In the WHQ scale aerobic training resulted also in lower scores for depressed mood and higher scores for attractiveness. Between-group differences in WHQ score change were significant in depressed mood. Changes in WHQ scores were dose-dependent in depressed mood, anxiety, and menstrual symptoms. Health-related quality of life was significantly improved in the areas of physical functioning and physical role limitation. Cardio-respiratory fitness improved, and lean muscle mass increased in the aerobic training group.

### Previous experimental studies

Earlier experimental studies concerning aerobic training and hot flushes are few; only two randomized controlled trials have included symptomatic women, whereas several other studies have included women based on age criteria (15). The first randomized study was performed in Sweden and reported promising results concerning exercise in spite of small sample size (24). In a larger (*n* = 164) 4-month trial the effects of yoga and moderate intensity walking on menopausal symptoms and mental health were assessed (25). Their results showed that both groups had decreases in hot flushes. Significant increases in positive affect scores were found in the walking and yoga groups but not among controls. In a Turkish study, 65 menopausal women participated in an aerobic exercise programme for 6 months (16), and the intervention group had significantly fewer hot flushes and other menopausal symptoms.

In our study, women in the intervention group reported a decreased frequency of hot flushes, especially during night-time. An increased level of aerobic training probably improved their quality of sleep and may have resulted in fewer awakenings due to nighttime hot flushes. Previous randomized studies have showed that adults with moderate sleep complaints can improve their sleep quality by moderate-intensity exercise programmes (26,27), but lower-intensity exercise such as walking or low-intensity yoga programmes may be ineffective (28). Decrease in daytime hot flushes was small, even if the downward trend was steeper among the intervention group until half of the intervention time; thereafter the trend was upward among both groups.

Previous intervention population studies have shown that increased physical activity has been associated with improvement of all dimensions of quality of life, but magnitude in improvement of mental health scale was smaller than in the physical health scale (29). In our results the improvements were found both in mental and in physical health items, possibly due to the sample characteristics, sedentariness and daily symptoms. A randomized, controlled trial involving 464 menopausal women showed quality of life (QoL) improvements after 6 months of regular exercise, independent of weight change (30). However, in the study the aim was not to relieve hot flushes. Another non-controlled trial for 16 weeks, aimed at increasing QoL by walking three sessions per week 45 minutes at a time, and involving 16 women, showed favourable results (31). In a study carried out by Teoman et al. (32), aerobic fitness and quality of life were improved after 6 weeks' training. Our results are parallel with these findings based on smaller samples, confirming the benefits of exercise both on health-related QoL and night-time hot flushes.

### Previous non-experimental studies

The results of some non-experimental studies have shown associations between exercise and lower rates of hot flushes and better QoL (15). An observational study by Aiello et al. (13) showed an increased severity of hot flushes in the exercise intervention group compared with controls. The women in the study were relatively old (mean age 60.6 years), and the majority of them reported symptoms other than hot flushes. Frequent physical exercise has been associated with a risk of greater severity and frequency of hot flushes in other non-experimental studies as well (10,11). In a multi-ethnic sample of 3,302 women in the Study of Women's Health in the Nation (SWAN), a trend of increasing prevalence of all symptoms with decreasing physical activity was found (31). In the study by Gold et al. (6) women were asked about their perception of activity level but not the type of activity. Thus, observational and experimental studies have conflicting results due to differences in assessment, i.e. questions concerning physical activity and sample characteristics such as size, age, and menopausal status of the women.

### Strengths

Strengths of our study include the trial design, use of valid quality of life scales, and inclusion of women with moderate to severe hot flushes. The trial design was comprehensive for evaluation of exercise efficacy as regards both hot flushes and menopausal QoL. Furthermore, the primary outcome of the study was occurrence of hot flushes, which was measured with a validated instrument (WHQ). According to a literature review of instruments used to assess health-related quality of life during and after menopause (19), both SF-36 and WHQ are reliable and may overlap in mental health areas. The effect was modest on menopausal health, while the benefits were more seen in general health, as shown in previous physical activity studies as well (29).

Generally, the non-response rate to the paper-based questionnaires was low. Non-adherers differed from adherent women in regard to age and weight—they were younger and lighter. The influence of these issues probably did not affect the primary outcome but might have had an effect on phone-based questionnaire results if imputation of the missing data was not performed.

In outcome assessment we used a new method of daily mobile phone contact. The method of daily responding decreases recall bias, and the questions were simple (yes/no). To our knowledge no previous study has involved the use of similar technology and mobile phone questionnaires in connection with menopausal symptoms. This novel technology enabled minimization of recall bias, since menopausal hot flushes were reported twice a day. Measurement of cardiorespiratory fitness was based on the UKK Walk Test, which has been widely used and is feasible for both scientific and practical purposes (17).

An additional strength of the study is a control group programme. The control group attended the lectures during the trial, since otherwise they might have been more likely to drop out from the trial. Thus, the main purpose of the lectures was to motivate and support the control group without aerobic training. Since both groups were given the same lectures, exercise training effect may be clearer than if the control group was not supported at all.

### Weaknesses

Regarding weaknesses of our study we acknowledge that the phone-based assessment of hot flushes was not based on a validated instrument. Although the frequency of hot flushes was accurately reported, more than 60% of the daily mobile phone responses lacked information regarding their severity, which was not compulsory information in the questionnaire. The majority of the women were unsure of the severity of their hot flushes and left the area blank. This is a limitation which should be taken into account in future mobile phone questionnaires. Before starting the trial the women kept 2-week diaries of daily symptoms by way of conventional questionnaires, not mobile phone diaries. In the feedback questionnaires after the intervention the women reported difficulties in judging the severity of symptoms during the mobile phone survey. In spite of these limitations, our study showed high adherence, since only 14 women in the intervention group and 8 in the control group dropped out.

As another limitation in our trial, the women were on average only slightly overweight, and all were of Caucasian origin. Therefore the results may not be generalized to populations with diverse ethnic backgrounds or a higher prevalence of obesity. However, possibly the effect of aerobic training could be at least as large among obese women, perhaps even larger.

Sample size calculations were based on a 30% difference between the groups in hot flushes. Based on this assumption and the expected prevalence of 68% of symptomatic women at base-line, we calculated that 154 women would be needed to have 80% power in the trial. However, in our study the difference between the groups at 6 months was less than the expected 30%. Therefore, the power of the study was limited to show differences in hot flushes.

### Future studies

The results of recent studies (33,34) have suggested that women with hot flushes may have adverse vascular changes. It is possible that symptomatic women may derive more benefit from aerobic training as regards both their general and vascular health, since aerobic training is beneficial for mental and physical health. Our aim was to study the efficacy of aerobic training on menopausal symptoms rather than effects on a general population; therefore we recruited sedentary, recently menopausal women. Further studies are needed to find out the most effective exercise (aerobic versus non-aerobic) among sufficiently active and fit women with menopausal symptoms. Also long-term compliance with exercise is a challenge to ours as well as to any study concerning exercise and health. Forthcoming studies need also to re-address the dose-response effect of exercise or other possible sham intervention. More experimental studies using mobile phone technology, different exercise training programmes, and fitness measurements are needed to confirm our findings in other populations.

## Conclusions

Aerobic training may decrease hot flushes, but more research is needed. Aerobic training improved meno-pausal and health-related quality of life among slightly overweight, symptomatic women.

## Trial registration

ISRCTN54690027, http://www.controlled-trials.com/

## References

[1] Pinkerton JV, Zion AS (2006). Vasomotor symptoms in menopause: where we've been and where we're going. J Women's Health.

[2] Tuomikoski P, Haapalahti P, Ylikorkala O, Mikkola TS (2010). Vasomotor hot flushes and 24-hour ambulatory blood pressure in recently post-menopausal women. Ann Med.

[3] Tuomikoski P, Haapalahti P, Sarna S, Ylikorkala O, Mikkola TS (2010). Vasomotor hot flushes and 24-hour ambulatory blood pressure in normotensive women: a placebo-controlled trial on post-menopausal hormone therapy. Ann Med.

[4] Santoro N (2008). Symptoms of menopause: hot flushes. Clin Obstet Gynecol.

[5] Rossouw JE, Anderson GL, Prentice RL, LaCroix AZ, Kooperberg C, Stefanick ML (2002). Writing group for the Women's Health Initiative investigators. Risks and benefits of the estrogen plus progestin use in healthy postmenopausal women: principal results from the Women's Health Initiative randomized controlled trial. JAMA.

[6] Gold EB, Sternfeld B, Kelsey JL, Brown C, Mouton C, Reame N (2000). Relation of demographic and lifestyle factors to symptoms in multi-racial/ethnic population of women 40–55 years of age. Am J Epidemiol.

[7] Huang AJ, Subak LL, Wing R, West DS, Hernandez AL, Macer J (2010). Program to Reduce Incontinence by Diet and Exercise Investigators. An intensive behavioral weight loss intervention and hot flushes in women. Arch Intern Med.

[8] Hammar M, Berg G, Lindgren R (1990). Does physical exercise influence the frequency of postmenopausal hot flushes?. Acta Obstetrica Gynecol Scand.

[9] Guthrie JR, Smith AM, Dennerstein L, Morse C (1994). Physical activity and the menopause experience: a cross-sectional study. Maturitas.

[10] Whitcomb BW, Whiteman MK, Langenberg P, Flaws JA, Romani WA (2007). Physical activity and risk of hot flushes among women in midlife. J Womens Health (Larchmt).

[11] Romani WA, Gallicchio L, Flaws JA (2009). The association between physical activity and hot flash severity, frequency and duration in mid-life women. Am J Hum Biol..

[12] Slaven L, Lee C (1997). Mood and symptom reporting among middle-aged women: the relationship between menopausal status, hormone replacement therapy and exercise participation. Health Psychol.

[13] Aiello EJ, Yasui Y, Tworoger SS, Ulrich CC, Irwin ML, Bowen D (2004). Effect of a yearlong, moderate-intensity exercise intervention on the occurrence and severity of menopause symptoms in postmenopausal women. Menopause.

[14] Wilbur JE, Miller AM, McDevitt J, Wang E, Miller J (2005). Menopausal status, moderate-intensity walking and symptoms in midlife women. Res Theory Nurs Pract..

[15] Daley AJ, Stokes-Lampard HJ, MacArthur C (2009). Exercise to reduce vasomotor and other menopausal symptoms: a review. Maturitas.

[16] Karacan S (2010). Effects of long-term aerobic exercise on physical fitness and postmenopausal symptoms with menopausal rating scale. Science & Sports.

[17] Oja P, Laukkanen R, Pasanen M, Tyry T, Vuori I (1991). A 2-km walking test for assessing the cardiorespiratory fitness of healthy adults. Int J Sports Med.

[18] Hunter M (2000). The Women's Health Questionnaire (WHQ): The development, standardization and application of a measure of mid-aged women's emotional and physical health. Qual Life Res.

[19] Zöllner YF, Acquadro C, Schaefer M (2005). Literature review of instruments to assess health-related quality of life during and after menopause. Qual Life Res.

[20] Aalto A-M, Aro S, Aro AR, Mähönen M (1995). RAND 36-item health survey 1,0 [Finnish validation study].

[21] Brooke J, Jordan PW, Thomas B, Weerdmeester BA, McClelland IL (1996). SUS: A quick and dirty usability scale. Usability evaluation in industry.

[22] Borg G (1970). Perceived exertion as an indicator of somatic stress. Scand J Rehab Med.

[23] Asikainen TM, Miilunpalo S, Oja P, Rinne M, Pasanen M, Uusi-Rasi K (2002). Randomized, controlled walking trials in postmenopausal women: the minimum dose to improve aerobic fitness?. Br J Sports Med..

[24] Lindh-Åstrand L, Nedstrand E, Wyon Y, Hammar M (2003). Vasomotor symptoms and quality of life in previously sedentary postmenopausal women randomized to activity of estrogen therapy. Maturitas.

[25] Elavsky S, McAuley E (2007). Physical activity and mental health outcomes during menopause: a randomized controlled trial. Ann Behav Med.

[26] King AC, Oman RF, Brassington GS, Bliwise DL, Haskell WL (1997). Moderate-intensity exercise and self-rated quality of sleep in older adults. A randomized controlled trial. JAMA.

[27] Tworoger SS, Yasui Y, Vitiello MV, Schwartz RS, Ulrich CM, Aiello EJ (2003). Effects of a yearlong moderate-intensity exercise and a stretching intervention on sleep quality in postmenopausal women. Sleep.

[28] Elavsky S, McAuley E (2007). Lack of perceived sleep improvement after 4-month structured exercise programs. Menopause.

[29] Wolin KY, Glynn RJ, Colditz GA, Lee IM, Kawachi I (2007). Long-term physical activity patterns and health-related quality of life in U.S. women. Am J Prev Med..

[30] Martin CK, Church TS, Thompson AM, Earnest CP, Blair SN (2009). Exercise dose and quality of life: results of a randomized controlled trial. Arch Int Med.

[31] Riesco E, Tessier S, Perusse F, Turgeon S, Tremblay A, Weisnagel J (2010). Impact of walking on eating behaviors and quality of life of premenopausal and early postmenopausal obese women. Menopause.

[32] Teoman N, Özcan A, Acar B (2004). The effect of exercise on physical fitness and quality of life in postmenopausal women. Maturitas.

[33] Tuomikoski P, Ebert P, Groop PH, Haapalahti P, Hautamäki H, Rönnback M (2009). Effect of hot flushes on vascular function: a randomized trial. Obstet Gynecol.

[34] Sternfield B, Wang H, Quesenberry CP, Abrams B, Everson-Rose SA, Greendale G (2004). Physical activity and changes in weight and waist circumference in midlife women: findings from the study of Women's Health Across the Nation. Am J Epidemiol.

